# The implementation of the SPAD-502 Chlorophyll meter for the quantification of nitrogen content in Arabica coffee leaves

**DOI:** 10.1016/j.mex.2024.102566

**Published:** 2024-01-11

**Authors:** Suwimon Wicharuck, Sutasinee Suang, Chatchawan Chaichana, Yupa Chromkaew, Nipon Mawan, Phonlawat Soilueang, Nuttapon Khongdee

**Affiliations:** a Office of Administration Research, Chiang Mai University, Chiang Mai 50200, Thailand; b Energy Technology for Environment Research Center, Faculty of Engineering, Chiang Mai University, Chiang Mai 50200, Thailand; c Department of Highland Agriculture and Natural Resources, Faculty of Agriculture, Chiang Mai University, Chiang Mai 50200, Thailand; d Department of Plant and Soil Science, Faculty of Agriculture, Chiang Mai University, Chiang Mai 50200, Thailand

**Keywords:** Arabica Coffee leaves, Highland agriculture, Leaf greenness, SPAD-502, Total nitrogen, Non-destructive nitrogen measurement in specific coffee crown level

## Abstract

The utilization of a non-destructive SPAD-502 chlorophyll meter, which enables the measurement of nitrogen status in plant leaves, has gained popularity in agronomic crops. Its application to horticultural crops like coffee remains relatively uncommon. The device provides quick and real-time measurements, helping to provide on-time nitrogen fertilizer to coffee plants before deficiency signs occur. Coffee leaves are characterized by thick and waxy leaves, together with many layers of tree crown. Therefore, the objective of this study was to develop a method for measuring nitrogen levels in coffee plants using the SPAD-502 Chlorophyll meter for an appropriate nitrogen fertilizer application rate in Arabica coffee plants.

• Coffee trees were separated into upper, middle and lower levels. Data on SPAD values and total nitrogen were analyzed.
• Pearson Correlation Coefficient (R), Coefficient of Determination (R^2^) and linear regression were calculated for different three levels of both SPAD-502 and total nitrogen values.
• The results revealed a strong correlation (R^2^ = 0.63) between the SPAD readings of coffee leaves obtained from the upper canopy and their nitrogen content.

These findings can provide a good concept of which coffee crown level will be a better part for measuring N content using a SPAD-502 Chlorophyll meter.

## Graphical abstract

**Figure:**
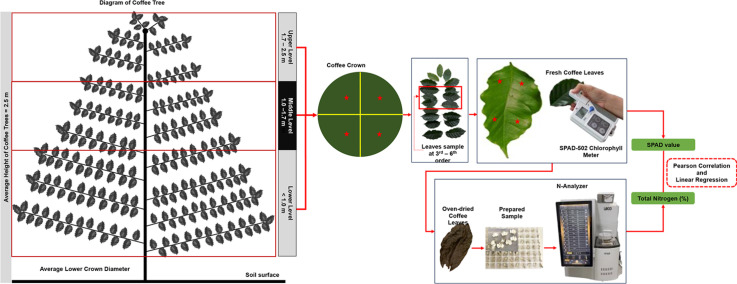

Specifications table

**Table:** 

Subject area:	Agricultural and Biological Sciences
More specific subject area:	*Nitrogen status measurement in coffee plant*
Name of your method:	*Non-destructive nitrogen measurement in specific coffee crown level*
Name and reference of original method:	SPAD-502 Chlorophyll meter *Kjeldahl method* *Nitrogen combustion method*
Resource availability:	*Data will be made available on request.*

## Method details

Coffee leaf nitrogen (N) level analysis for an optimum fertilizer application to coffee trees at the right time has been widely recommended in many investigations. A conventional practice is to collect coffee leaves by counting branches from the uppermost point of the vertical stem until reaching the 8th to 12th lateral branch and choosing the most recently developed leaf from these side branches, typically located around the 3rd or 4th pair of leaves from the tip of the branch. Then, selected leaves that have reached their maximum size and show similar color and texture traits as the elder leaves on the coffee plant, and analyzed in the laboratory. The laboratory analysis has the potential to provide precise results; however, it is characterized by a significant investment of time and resources. The results from the laboratory somehow cannot provide real-time information to the farmers for maintaining nutrient management for the crops. Therefore, another non-destructive method has been developed for rapid measurement based on the correlation between leaf chlorophyll and nitrogen (N) concentrations. This is due to the fact that chloroplasts within leaf cells hold approximately 70 % of the nitrogen in leaves and the nitrogen status in leaves is resulted in leaf greenness [1], [2], [3]. The leaf characteristics of agronomic crops are typically green and smooth, while horticultural crops are varied. Real-time nitrogen evaluation in coffee leaves using the SPAD-502 chlorophyll meter can help to determine the nitrogen status for optimum fertilizer application. Coffee leaves are characterized by thick and waxy leaves that are different from those of agronomic crops. No specific recommendation of the appropriate canopy level for measuring nitrogen levels using a SPAD-502 in published literature [4], [5], [6]. Most recommendations primarily suggest the specific leaf positions that should be sampled.

Therefore, our idea was to develop a proper and easy method of using the SPAD-502 Chlorophyll meter [2] to measure the level of greenness in coffee leaves by focusing on different sections of the tree crown. The output of this study can enhance the precision of determining the ideal N fertilizer levels for coffee trees and can offer comprehensive insights for various segments of the tree canopy. This innovative approach seeks to revolutionize the traditional labor-intensive method by offering a non-destructive, cost-effective, and real-time method of assessing leaf greenness. This empowers coffee producers to make well-informed and prompt choices about nitrogen fertilization. The implementation of the SPAD-502 Chlorophyll meter shows potential in streamlining the procedure while guaranteeing precise and effective nitrogen management in coffee farming.

*Developing of total nitrogen measurement using non-destructive SPAD-502 chlorophyll meter*

The method consisted of three main parts as follows:

1. Measurement of SPAD values in the field

**Fig. 1:**
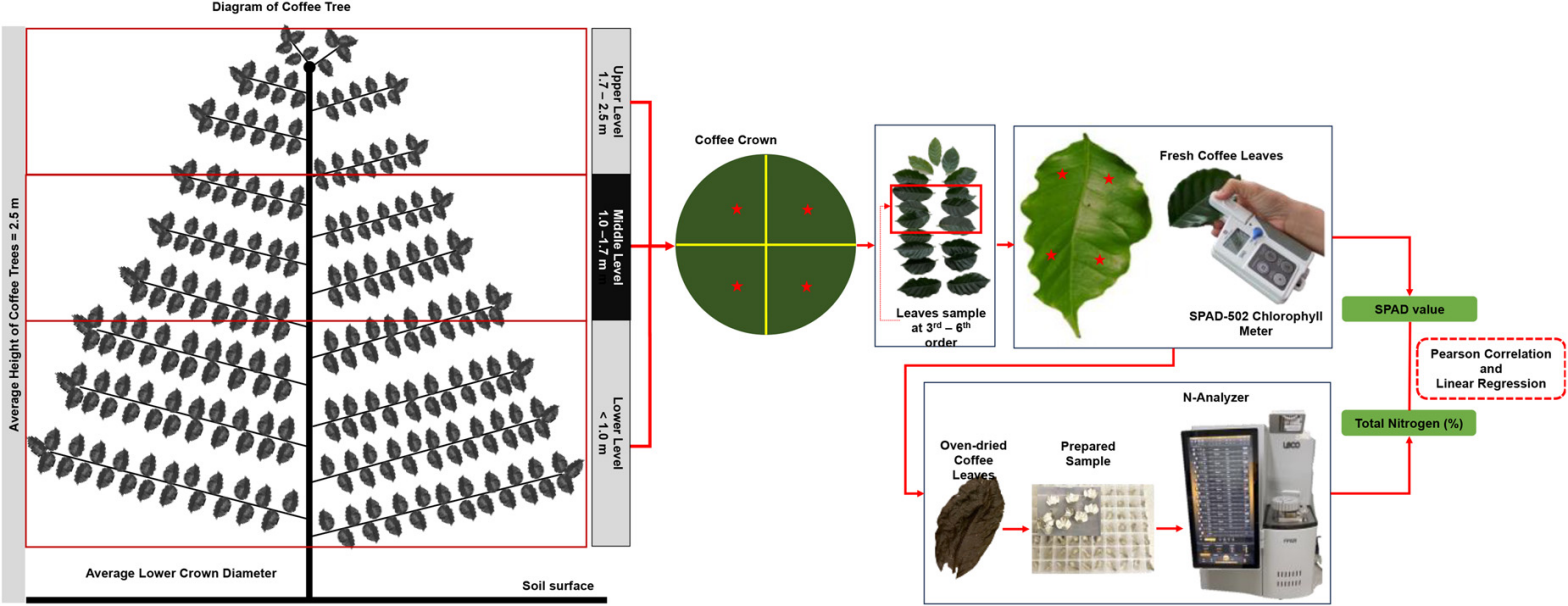
A diagram of SPAD-502 chlorophyll meter measurement and total nitrogen combustion method.

Five coffee trees (approximately 20 years old) were randomly selected for data collection. Each selected coffee tree was divided into 3 levels: upper, middle, and lower levels (Fig. 1). The average height of each section was 0.8 m in the upper level, 0.7 m in the middle level, and 1 m in the lower level. The coffee crown was further divided into four positions within each level. Three coffee leaves between the 3rd and 6th were then randomly sampled and four points of each coffee leaf were measured for leaf greenness using the SPAD-502 chlorophyll meter (Konica Minolta) in each position. The total points were 240 points per level; then, these SPAD values were averaged for each position.

2. Total nitrogen analysis by combustion method

The leaf samples were then collected for total nitrogen analysis using the combustion method at the Central Laboratory, Faculty of Agriculture, Chiang Mai University. The coffee leaf samples were prepared before laboratory analysis. The samples were placed in the oven at 65 °C for three days. The oven-dried samples from each position were mixed and then finely ground (total ground samples were 20 samples per level). About 0.10–0.15 g of ground samples were encapsulated within N-free tin foil and placed in the N-Analyzer (LECO). The total concentration of nitrogen was then expressed as a percentage.

3. Method calibration

**Fig. 2:**
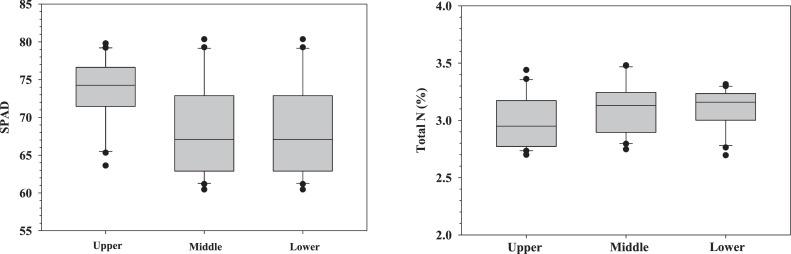
Box plot of SPAD values and total nitrogen (%) at different three levels (upper, middle and lower). The lowest and highest boxes represented the 25th and 75th percentiles, respectively. The line inside the box represented the median values of the data set. The lowest lines showed the smallest non-outlier values, while the highest lines displayed the maximum non-outlier values of the data set.

The Pearson correlation coefficient (R) between SPAD values and total nitrogen content was estimated. Finally, the coefficient of determination (R^2^) was calculated and regression analysis was evaluated for different three levels. Table 1 and Fig. 2 showed SPAD values and total N at different levels. The highest SPAD values were observed at the upper level (73.56) in comparison to the other levels, with the values being 68.68 at the middle and 67.18 at the lower levels. An opposite trend was found on the total N values, as the lowest values occurred at the upper level (2.98 %), followed by the middle and lower levels (3.10 %). The SPAD value is always correlated to the leaf greenness, therefore, this can be implied that the upper part of coffee trees will be a good area for SPAD measurement.

**Table 1:** Average and standard deviation (SD) of SPAD values and total nitrogen (%) under three different levels. N = number of investigations.

Parameters	Position	Average ± SD
**SPAD (*N*** **=** **20)**	Upper	73.56±4.57
	Middle	68.68±6.21
	Lower	67.18±5.00
**Total Nitrogen (%) (*N*** **=** **20)**	Upper	2.98±0.23
	Middle	3.10±0.22
	Lower	3.10±0.17

**Fig. 3:**
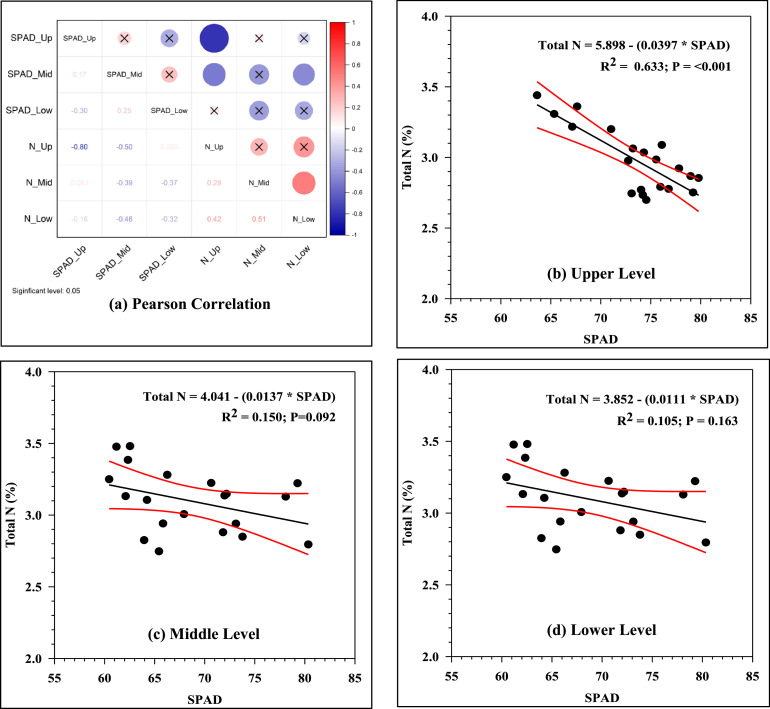
(a) Pearson Correlations between SPAD and Total N at three different levels. SPAD was the greenness values measured by SPAD-502 Chlorophyll meter and N displayed total nitrogen in leaves analyzed by combustion method. Up, Mid and Low referred to the upper, middle and lower levels. (b)-(d) Linear Regression between SPAD and Total N (%) at three different levels.

The Pearson correlation coefficient (R) showed negative correlations between SPAD and total N values for three levels. Only the upper level displayed a significant correlation of those two values at *R* = −0.80, while the middle and lower levels had very low values of Pearson correlation as *R* = −0.39 at the middle level and *R* = −0.32 at the lower level (Fig. 3a). This also indicated that higher values of SPAD did not always correspond to higher values of total nitrogen in plant leaves. In addition, the regression coefficient of determination (R^2^) was significantly correlated at the upper level (R^2^ = 0.633 at *P* < 0.001), but it was non-significant at the middle and lower levels (R^2^ = 0.150 at *P* = 0.092 at the middle level and R^2^ = 0.105 at *P* = 0.163 at the middle level), as shown in Fig. 3b-d. The results confirmed that the upper part can be a good zone for SPAD measurement in the field.

## Conclusion and future work

This study displays the capacity of the SPAD-502 Chlorophyll meter as a non-invasive and immediate method for evaluating nitrogen levels in coffee leaves. This research demonstrated the association between nitrogen content and SPAD values by using the SPAD-502 Chlorophyll meter on various sections of the coffee tree crown.

Using SPAD-502 to determine the nitrogen level in coffee leaves could ideally be done at the upper canopy level, with the linear regression of total *N* = 5.898 – (0.0397 × SPAD value) and R^2^of more than 0.6.

To use this method in the fields, it is necessary to measure the greenness of the leaves at an average height of 1.7 - 2.5 m. Choose four branches that have coffee leaves that are relatively green. Next, three leaves are selected from each branch in the 3rd to 6th order, and the coffee leaf greenness values must be measured at a minimum of four spots on each leaf.

## CRediT authorship contribution statement

**Suwimon Wicharuck:** Conceptualization, Methodology, Writing – review & editing, Visualization, Validation. **Sutasinee Suang:** Data curation, Writing – original draft. **Chatchawan Chaichana:** Conceptualization, Supervision. **Yupa Chromkaew:** Writing – original draft, Investigation, Validation. **Nipon Mawan:** Writing – original draft, Investigation, Validation. **Phonlawat Soilueang:** Data curation, Writing – original draft. **Nuttapon Khongdee:** Conceptualization, Methodology, Writing – review & editing, Visualization, Validation.

## Declaration of competing interests

The authors declare that they have no known competing financial interests or personal relationships that could have appeared to influence the work reported in this paper.

## References

[1] Madakadze I.C., Stewart K.A., Madakadze R.M., Peterson P.R., Coulman B.E., Smith D.L. (1999). Field evaluation of the chlorophyll meter to predict yield and nitrogen concentration of switchgrass. J. Plant Nutr..

[2] Minolta C. (1989).

[3] Wood C.W., Tracy P.W., Reeves D.W., Edmisten K.L. (1992). Determination of cotton nitrogen status with a handheld chlorophyll meter. J. Plant Nutr..

[4] Mielke M.S., Schaffer B. (2010). Photosynthetic and growth responses of Eugenia uniflora L. seedlings to soil flooding and light intensity. Environ. Exp. Bot..

[5] Putra B.T.W., Soni P., Morimoto E., Pujiyanto P. (2018). Estimating biophysical properties of coffee (Coffea canephora) plants with above-canopy field measurements, using CropSpec®. Int. Agrophys..

[6] Widjaja Putra B.T., Soni P. (2018). Dataset of chlorophyll content estimation of Coffea Canephora using Red and Near-Infrared consumer-grade camera. Data Brief.

